# Primary osteogenic sarcoma of the breast: A case report

**DOI:** 10.1186/1757-1626-1-148

**Published:** 2008-09-10

**Authors:** Shabuddin Khan, Ewen A Griffiths, Nigam Shah, Srinivasan Ravi

**Affiliations:** 1 Department of General Surgery, Blackpool Victoria Hospital, Blackpool Fylde and Wyre NHS Trust, Blackpool, FY2 8NR, UK; 2 Department of Histopathology, Blackpool Victoria Hospital, Blackpool Fylde and Wyre NHS Trust, Blackpool, FY2 8NR, UK

## Abstract

**Introduction:**

Primary osteosarcoma of the breast is a rare malignant tumour. It is typically a poor prognosis tumour, which has some interesting features worthy of discussion. We report a case of primary osteosarcoma of the breast and summarise the previous medical literature to highlight several details of this unusual tumour.

**Case presentation:**

A 66-year-old Caucasian lady presented with painless lump in her right breast. Mammography showed features of fibroadenoma with calcification and fine needle aspiration cytology was reported as showing malignant cells (C5). Wide local excision was performed and histological features were consistent with primary osteosarcoma with predominance of osteoclastic activity. Subsequent completion mastectomy was performed because of suspicion of incomplete excision. She remains disease free 8 years from her initial surgery.

## Introduction

Extra-osseous osteogenic sarcomas have been reported from the thyroid gland, kidney, bladder and soft tissues, but mammary osteogenic sarcomas constitute only a small group [1]. Primary sarcomas of the breast are extremely rare and make up less than 0.1% of all malignant tumours of the breast [2]. Fewer than 150 cases of primary osteosarcoma of breast have been reported in the medical literature in the last 50 years. When this tumour develops in the breast, it originates either from normal breast tissue *de novo*, or as metaplastic differentiation of a primary benign or malignant breast lesion. Secondary deposits from a primary bone sarcoma occur only rarely. Primary osteogenic breast cancer is usually considered a poor prognosis tumour, with high risk of disease recurrence and haematogeneous spread, most commonly to the lungs.

We present the case of a 66 year old Caucasian woman who was diagnosed with an osteogenic sarcoma of the breast and has survived eight years from surgical resection. We have performed a thorough literature review and would like to use this case to highlight several details of this unusual tumour.

## Case presentation

A 66-year-old Caucasian lady presented with a painless, enlarging lump in her right breast. There was no history of trauma, skin changes, nipple discharge, or any other breast lumps. She was otherwise asymptomatic with no symptoms of bone pain. There was no family history of breast cancer. She had a past medical history of severe chronic obstructive airways disease, previous fracture neck of femur and surgery for a hiatus hernia. There was no prior history of radiotherapy. Clinical examination revealed a non-tender hard lump in right upper quadrant with no palpable axillary lymph nodes. Mammography showed features of fibroadenoma with calcification. Fine needle aspiration cytology (FNAC) was positive for malignant cells (C5).

**Figure 1:**
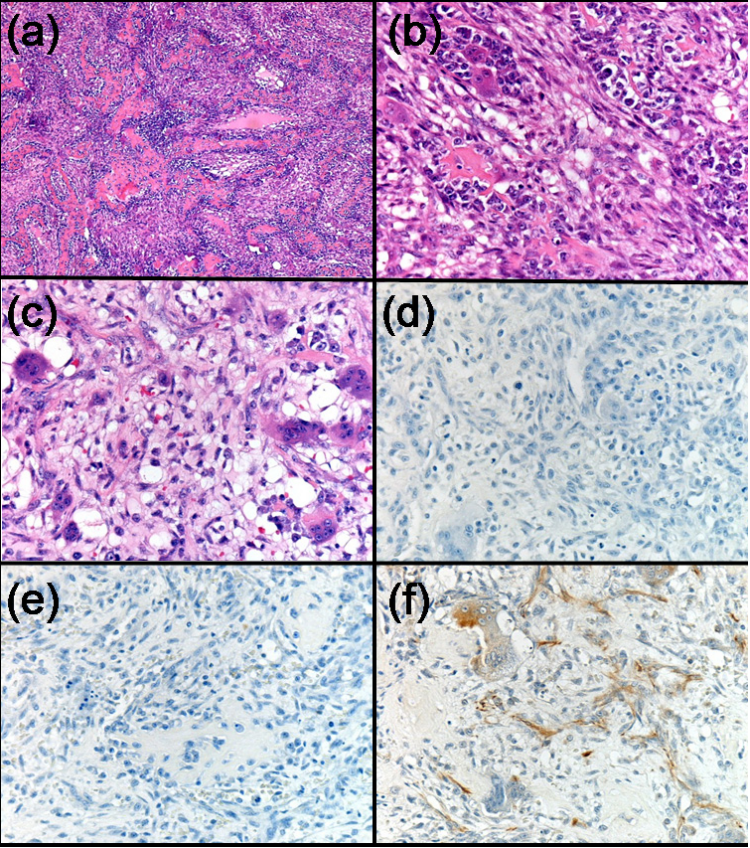
(a) Osteosarcoma (×4); (b) Giant cells with osteoid formation (×20); (c) Giant cells with osteoid formation (×20); (d) Negative immunohistochemical staining for CAM5.2 (×20); (e) Negative immunohistochemical staining for MNF116 (×20); (f) Positive immunohistochemical staining for vimentin (×20).

Wide local excision of the breast lump was performed in August 1998. This was performed under local anaesthesia due to her poor respiratory function. Macroscopic examination of the excised specimen revealed it to be composed of a piece of fibrofatty tissue measuring 6 × 4 × 2 cm that weighed 33 grams. There was a 2 cm cystic mass containing a necrotic tumour. The tumour appeared to infiltrate the cyst wall and extended to within 3 mm from the nearest resection margin. Microscopically, an encapsulated tumour composed of spindle cells, osteoclastic giant cells and osteoblasts was found. The spindle cells contained plump nuclei with prominent nucleoli. The cytoplasm was pale to eosinophilic with indistinct cell border. The tumour showed high apoptotic activity that was mitotically active with 24 mitoses/10 high power field. The tumour demonstrated osteoclastic and osteoblastic activity with marked osteoid formation (Figure 1). Immunohistochemistry was performed and the tumour cells were positive for vimentin. MNF116 and CAM5.2 (which are broad spectrum cytokeratin epithelial markers) staining was negative proving this was a primary osteosarcoma rather than metaplastic breast sarcoma (Figure 1). The tumour was also negative for S100, desmin and smooth muscle actin. The tumour infiltrated the breast capsule and extended very close to the resection margins, therefore it was not possible to be certain that excision was complete and further excision was advised.

Computer tomography of the thorax and abdomen showed no evidence of distant metastasis. The patient was optimised for general anaesthesia after consultation with a Consultant Respiratory Physician who managed to improve her respiratory function with medical therapy. Subsequent right sided mastectomy was performed under general anaesthesia in January 1999. As the spread of extraskeletal osteosarcoma is haematogenous rather via lymph, axillary lymphadenectomy was not perfomed. Histology of the mastectomy specimen showed no evidence of residual tumour involvement.

She was followed up in the outpatient clinic every 6 months. In March 2001, a magnetic resonance imaging (MRI) scan of right axilla was performed for a clinical suspicion of an axillary lump, but this was found to be normal. She was last reviewed in the breast clinic in November 2006 and was found to have no clinical evidence of recurrent disease and was therefore discharged back to the care of her General Practitioner.

## Discussion

Extra-skeletal sarcomas tend to occur in the over 50 year old age group [3], which is in contrast to osteogenic sarcoma arising from bone which mainly occur in children and adolescents. Primary osteosarcomas of the breast are very rare and account for less than 0.1% of breast tumours [2] and are often found in women with a mean age of 64 years [4].

The carcinogenesis of primary osteogenic sarcoma of the breast is not clear, but an origin from totipotent mesenchymal cells of the breast stroma or a transformation from a pre-existing breast lesion has been suggested. For example, the tumour may originate from either benign breast neoplasms, such as a long standing fibroadenoma or an intraductal papilloma; or a malignant breast lesion, such as a phyllodes tumour [5]. To confirm the diagnosis of primary osteogenic sarcoma of the breast, the direct extension of an osteogenic sarcoma arising from the ribs or sternum should be excluded. Primary breast carcinoma with extensive osseous metaplasia must also be considered [6].

Preoperative diagnosis is unusual and most patients the correct histological diagnosis of only established after surgical resection[7]. The mammographic appearances of these tumours are of a well circumscribed dense lesion within the breast tissue with focal or extensive coarse calcifications [8]. The border may be regular or irregular. The mammographic appearances may be deceptively benign and may imitate a benign fibroadenoma in a third of cases [4]. Fine needle aspiration cytology may not yield some clues to the diagnosis with features such as hypocellular or hypercellular smears with pleomorphic cells; scarce or abundant metachromatic amorphous material, suggestive of osteoid; osteoclast-like giant cells; and stromal fragments [9].

The main histological differential diagnosis in our case was metaplastic carcinoma [2], however this was excluded because of negative epithelial markers on immunohistochemical staining. Metaplastic carcinoma is recognised either by the presence of carcinomatous compenent on H&E staining or by cytokeratin immunoreactivity of the neoplastic spindle cells [2]. Phyllodes tumour was not a differential diagnosis in our case, but would be it in any sarcoma with a predominantly fibroblastic component. However, our case had characteristic osteoid formation and osteoclastic giant cells and it is possible to say with conclusively that our case is an osteosarcoma (Figure 1).

Surgical management of these tumours is either by wide local excision or mastectomy depending on the size of the tumour and remaining breast tissue. It is important to achieve a complete resection with negative resection margins, as margin status is a major factor for local disease recurrence. Axillary clearance is not necessary as these tumours do not spread via the lymphatic route. Adjuvant radiotherapy to the chest wall may reduced the risk of local recurrence [10]. Although adjuvant chemotherapy with either doxorubicin, cisplatin or ifosfamide based regimes has increased the survival of primary bone sarcomas, there are no proven benefits to the use of these regimes in primary osteosarcoma of the breast.

The long-term prognosis is uncertain due to the small number of cases reported in the medical literature. Silver et al reported a 5 year survival of 38% in a study of 50 patients with primary breast oestosarcoma [4]. Twenty eight percent of patients developed local recurrence and 41% distant metastases. Haematogenous metastases most commonly occur to the lungs (80%), bone (20%), and liver (17%). Prognostic factors included tumour size, number of mitoses, presence of stromal atypia, histological subtype and resection margin involvement [4].

The learning points from our case are presented in Table 1.

**Table 1:** Learning points from this case report

	**Learning points**
1.	Primary breast sarcomas are rare tumours of the breast. They make up less than 0.1% of all breast tumours.
2.	Tumour development is either from normal breast tissue *de novo*, or as metaplastic differentiation of a primary benign or malignant breast lesion. Secondary deposits from a primary bone sarcoma occur only rarely, but need to be
3.	excluded clinically. Diagnosis is made by careful assessment of the histological specimen, together with immunohistochemical staining.
4.	Typically primary osteogenic breast cancer is usually considered a poor prognosis tumour, with high risk of disease recurrence and haematogeneous spread, most commonly to the lungs. Lymph node metastases do not occur.

## Consent

Written informed consent was obtained from the patient for publication of this case report and accompanying images. A copy of the written consent is available for review by the Editor-in-Chief of this journal.

## Competing interests

The authors declare that they have no competing interests.

## Authors' contributions

SK wrote the draft of the manuscript and performed the literature search; EG helped performed the literature research and revised the manuscript; NS re-examined the surgical specimen, took the photomicrographs of the specimen and helped with revising histopathological aspects of the manuscript; SR performed the surgical procedure and revised the manuscript for intellectual content.
